# VLSI Design of Trusted Virtual Sensors

**DOI:** 10.3390/s18020347

**Published:** 2018-01-25

**Authors:** Macarena C. Martínez-Rodríguez, Miguel A. Prada-Delgado, Piedad Brox, Iluminada Baturone

**Affiliations:** Instituto de Microelectrónica de Sevilla IMSE-CNM, CSIC, Universidad de Sevilla, Américo Vespucio, 41092 Sevilla, Spain; prada@imse-cnm.csic.es (M.A.P.-D.); brox@imse-cnm.csic.es (P.B.); lumi@imse-cnm.csic.es (I.B.)

**Keywords:** virtual sensors, CMOS integrated circuits, data security, hardware security, Physical Unclonable Function (PUF), piecewise linear approximation

## Abstract

This work presents a Very Large Scale Integration (VLSI) design of trusted virtual sensors providing a minimum unitary cost and very good figures of size, speed and power consumption. The sensed variable is estimated by a virtual sensor based on a configurable and programmable PieceWise-Affine hyper-Rectangular (PWAR) model. An algorithm is presented to find the best values of the programmable parameters given a set of (empirical or simulated) input-output data. The VLSI design of the trusted virtual sensor uses the fast authenticated encryption algorithm, AEGIS, to ensure the integrity of the provided virtual measurement and to encrypt it, and a Physical Unclonable Function (PUF) based on a Static Random Access Memory (SRAM) to ensure the integrity of the sensor itself. Implementation results of a prototype designed in a 90-nm Complementary Metal Oxide Semiconductor (CMOS) technology show that the active silicon area of the trusted virtual sensor is 0.86 mm${}^{2}$ and its power consumption when trusted sensing at 50 MHz is 7.12 mW. The maximum operation frequency is 85 MHz, which allows response times lower than 0.25 μs. As application example, the designed prototype was programmed to estimate the yaw rate in a vehicle, obtaining root mean square errors lower than 1.1%. Experimental results of the employed PUF show the robustness of the trusted sensing against aging and variations of the operation conditions, namely, temperature and power supply voltage (final value as well as ramp-up time).

## 1. State of the Art

A virtual sensor estimates the value of a variable that is very difficult or costly to measure physically by modelling the relation between that variable and others that can be measured easily with low-cost commercial sensors. The use of virtual sensors has increased continuously since the early 1980s in a wide number of industrial applications, such as building monitoring [1], robotics [2], process control [3,4], or automotive engineering [5]. In the latter case, for example, virtual sensors are employed to monitor vehicle and driving status as well as road conditions and even communication between vehicles [5,6,7]. The model that relates input and output variables can be derived from fundamental physical laws using adjustable parameters, from empirical data of the input and output variables without any knowledge of the physical process (usually referred to as black-box models), or a combination of physical and empirical knowledge (a gray-box model). Neural networks and fuzzy logic techniques are used widely to obtain black- and gray-box models [6,7]. PieceWise-Affine (PWA) virtual sensors are also employed to provide black-box models [8,9,10].

Virtual sensors are usually implemented as software installed in the electronic control units [6,7,8]. However, faster responses, smaller sizes and lower power consumption are achieved if virtual sensors are implemented in hardware. Solutions that employ PWA Simplicial (PWAS) models implemented in Field Programmable Gate Arrays (FPGAs) were proposed in [9,10]. PWAS-based virtual sensors are simpler to implement in FPGAs than neural-network-based sensors and provide higher computation speed, as shown in [10]. FPGA implementations of PWA functions based on hyper-rectangular partitions (PWAR-based models) are further simpler than PWAS-based models, as shown in [11]. Taking into account that the sensor market size is growing (in the automotive industry, for example, approximately 22 billion sensors are estimated to be used per year by 2020 [12]), a CMOS Integrated Circuit (IC) solution for virtual sensors is very interesting since it can provide a minimum unitary cost with high performance in terms of area, power and speed. This is why this paper focuses on virtual sensor design into ICs. PWAR-based models are selected since they are very suitable to be realized by programmable ICs that can be adjusted to different applications.

Considering that virtual sensors are usually part of ubiquitous and distributed networks, security is becoming increasingly important [13,14]. The sensing data must be trusted by the receiver, hence the integrity of the output data must be ensured. However, the data can be authenticated by an impostor sensor. The counterfeit problem is of such magnitude that the Semiconductor Industry Association (SIA) keeps an anti-counterfeiting task force [15]. The trusted sensing significance has increased up to the point that it is considered not only by the sensors [14,16,17] but also by well-established protocols that interconnect them [18]. To solve the problem of the IC integrity, the key is not stored in the IC. It is recovered by using a Physical Unclonable Function (PUF) inside the sensor hardware [19]. Consequently, the trusted sensor can recover the cryptographic key, while any impostor sensor is unable to do that. To solve the problem of data integrity, Message Authentication Codes (MACs) are usually employed. MACs can be obtained from block ciphers (such as AES-CMAC) or from hash functions (such as HMAC). In the case of AES-CMAC, the block cipher AES encrypts the data and then AES-CMAC is applied to the resulting ciphertext to generate the authentication tag. Due to the significance of the standardization in any industrial application the standard Keyed-Hash Message Authentication Code (HMAC) was selected in a previous work [20]. The HMAC standard is essentially a two-pass hash-based MAC [21]. In [20], a PHOTON-based HMAC was implemented [22]. In this paper a new solution based on the authenticated encryption algorithm called AEGIS [23] is proposed. AEGIS algorithm is one of the third-round candidates of the Competition for Authenticated Encryption: Security, Applicability, and Robustness (CAESAR) [24]. It is recommended for lightweight solutions, providing small response times because symmetric key encryption and MAC are combined efficiently to share part of the computation. AEGIS not only authenticates the virtual sensor data but also encrypts it, ensuring confidentiality.

This paper describes the VLSI design of trusted virtual sensors. The paper is organized as follows. Firstly, the main features of the proposed trusted virtual sensor are described in Section 2. Then, an architectural description of the sensor is provided in Section 3. In Section 4, implementation results of the proposal in a 90-nm CMOS technology are shown and an application example in the automotive domain is described. Finally, conclusions are given in Section 5.

## 2. Features of the Proposed CMOS Sensor

The proposed sensor provides the encrypted value of a variable that is virtually measured. In addition, it provides an authentication code that ensures the integrity, confidentiality, and authenticity of the sensor data.

The virtual measurement is estimated from a PWA-based model. Both PWAR and PWAS forms are able to approximate any function and extract any black-box model. The PWAS form has been widely explored for virtual sensors [9,10]. However, the PWAR form is selected herein since its implementation is simpler than PWAS implementation.

The algorithm AEGIS is selected to encrypt and authenticate the virtual measurement providing confidentiality and authenticity to the virtual sensor. AEGIS offers a high security since it is not possible to recover the state and the key faster than exhaustive key search provided that a non-reused nonce is used and assuming that forgery attacks are not successful. The output provided by the proposed trusted virtual sensor are the used nonce, the resulting ciphertext, and the authentication tag.

The integrity of the virtual sensor is ensured if the key employed by AEGIS is not stored but recovered whenever needed by using PUFs. The trusted sensor is able to recover the cryptographic key shared with the receiver of the sensing data, while any impostor is unable due to the uniqueness provided by the start-up values of the SRAM in the sensor, which is exploited as a PUF. Non-sensitive Helper Data, *H*, are stored to recover the key with a Helper Data Algorithm (HDA) based on an Error Correcting Code (ECC) [25]. Helper Data do not reveal anything about the cryptographic key because the start-up values of SRAM cells obfuscate it. Similarly, Helper Data do not reveal anything about the intrinsic nature of the sensor because the cryptographic key obfuscates it.

Hence, two main components are differentiated in the proposed trusted virtual sensor. One part is associated with the generation of the virtual measurement and the other one provides the security of the measurement. They are both detailed in the following.

### 2.1. Virtual Sensing Based on PWAR approach

A black-box model establishes the relation between input variables and the empirical or simulated output. The virtual sensor is obtained using a black-box identification algorithm by assuming that the virtually measured output, *y*, is set as a PWAR function of the input variables, $x=\{x_{1},\ldots ,x_{n}\}$.

A generic PWA function with multiple inputs and one output $y\left(x\right):D\subset R^{n}\to R$ is represented as

(1) $$\begin{array}{c} y\left(x\right)=\sum_{j=1}^{n}f_{ij}\cdot x_{j}+f_{i0}\;\forall \;x\in P_{i}\left(i=1,\ldots ,P\right) \end{array}$$

where $f_{i}=\left[f_{i0}\cdots f_{in}\right]\in R^{n+1}$, and $P_{i}\subset D$ are *P* non-overlapping regions ($P_{i}\cap P_{j}=\emptyset \;\forall i\neq j$), called polytopes, which form a polyhedral partition of the domain, *D*, so that ${⋃}_{i=1}^{p}\;P_{i}=D$.

**Figure 1:**
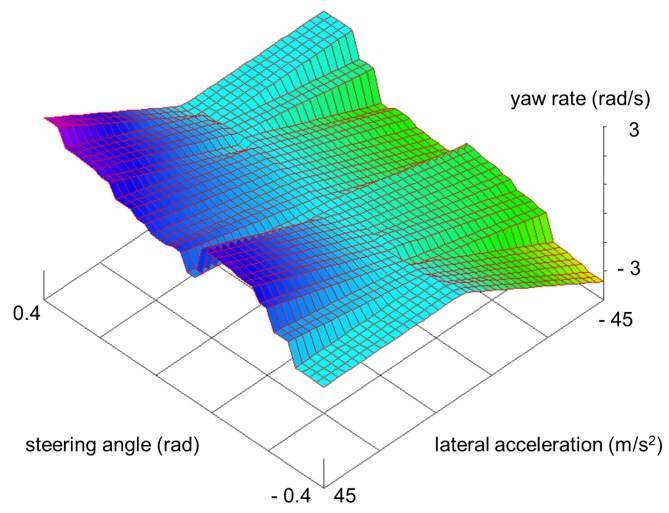
Vehicle yaw rate estimation by a PWAR-based virtual sensor.

In the case of regular PWAR functions, the domain is partitioned into hyper-rectangular polytopes by dividing each *k* dimension of the domain into $L_{k}=2^{p_{k}}$ intervals with the same amplitude, thus resulting $P=\prod_{k=1}^{n}L_{k}=\prod_{k=1}^{n}2^{p_{k}}=2^{\sum_{k=1}^{n}p_{k}}$ polytopes. Hence, a PWAR function is defined by its partition, $L=\{p_{1},\ldots ,p_{n}\}$, and the coefficients and the offset of each affine function, $F=\{f_{1},\ldots ,f_{P}\}$ with $P=2^{\sum_{k=1}^{n}p_{k}}$. Figure 1 shows a bi-dimensional example of a regular PWAR function with its domain partitioned into $32=16\times 2=2^{4}\times 2^{1}$ rectangles, that is L={4,1}.

The partition and affine functions of a PWAR function can be adjusted conveniently to approximate the relation between input variables and sensing variable to estimate. An algorithm has been developed to find the parameters {L,F} that minimize the mean square error (MSE) between the PWAR function output (variable to sense, *y*) and the empirical or simulated output data, $y_{\mu }$. The algorithm takes into account the constraints imposed by digital implementations, which are the following.

- Maximum number of input variables: *n*.
- Maximum number of hyper-rectangles: $P=2^{p}$.
- Maximum number of intervals per input: $Q=2^{q}$, being $L_{k}=2^{p_{k}}$ the number of intervals per input, so that, $q\geq p_{k}$ ∀k.

The algorithm determines the hyper-rectangular partition of the input domain, $L=\{p_{1},\ldots ,p_{n}\}$, and the value of the affine functions, $F=\{f_{1},\ldots ,f_{P}\}$.

First, the partition is established, and then an off-line optimization algorithm extracts the *F* parameters using a set of simulated or empirical values of the output and its corresponding input data. All input data should belong to the domain *D*. At least, a given percentage, *C*, of the hyper-rectangles in a partition should be covered by the data in order to proceed with optimization. Otherwise, such high resolution is not required and the partition is not considered. The result of the approximation algorithm for a given partition, *L*, is not only the value of the *F* parameters but also the MSE between the simulated or empirical output data and the output of the PWAR function (the MSE taking into account training and testing data). Then, the MSE of all possible partitions are compared, and the partition with the lowest MSE (with the MSE that allows the best trade-off taking into account training and testing data) is selected.

The algorithm steps are detailed in the pseudo-code of Algorithm 1. Its inputs are a set, *B*, of output data, $y_{\mu }$, with μ=1,…,M, corresponding to the input data $\left\{x_{1\mu },\ldots ,x_{n\mu }\right\}$; the value of *p* that fixes the maximum number of hyper-rectangles; the value *q* that fixes the maximum number of intervals per input; and the minimum percentage, *C*, of hyper-rectangles that should be covered by input data. Data set B is divided into two subsets, one used as training file and the other as test (validation) file.

The partitions with high resolution, that is, which verify that $p=\sum_{k=1}^{m}p_{k}$, are firstly explored with the function *TryPartition* (line 1.17) because they have higher capability of providing a lower approximation error (and they can be implemented in the proposed IC sensor as well as partitions with lower resolution). The function *TryPartition*(*A*, *B*, *C*) evaluates if the partition, *A*, meets or not the condition to be included in the partition list, Pool. The condition is that the data set, *B*, should belong to no less than the *C* in percentage of the hyper-rectangles of the partition, *A*. Otherwise, such high resolution model has too many undefined affine functions. Finally, the partition list is explored (with the function *Optimization*) to determine the partition that provides the lowest approximation error. The function *Optimization*(i,B) (line 1.34) applies Levenberg-Marquardt algorithm to find the parameters *F* that achieve the minimum MSE (and, hence, the Root Mean Square Error, RMSE) for each partition candidate, *i*, considering the data set *B* [26]. Since the partition candidate (hyper-rectangle) is fixed, this optimization finds the best linear (affine) regression for the data. The *F* parameters of hyper-rectangles not covered by data are fixed as the arithmetic mean of the *F* in the neighbourhood. Once the algorithm finishes, if all the coefficients, $f_{ij}$, associated with the input $x_{j}$ (i=1,…,P), are zero, that input can be removed, thus resulting $x=\{x_{1},\ldots ,x_{m}\}$ with m≤n.

The proposed sensor can be configured to provide several PWAR functions depending on the application. Therefore, it can be used to sense different variables. The configuration data are the number of input variables, and the number of intervals in which each dimension is divided that is, the hyper-rectangular partition is configurable. The sensor is also configurable in the affine functions of each hyper-rectangle by changing the value of the parameters associated with each hyper-rectangle. The input variables can be a same variable measured at several sampling times as well as the output measured at previous instants, for example, x={α[k]α[k−1]β[k]y[k−1]}.

**Algorithm 1** Pseudo-code of PWAR virtual sensor algorithm.**Require:**
$B=\left[x_{11},\cdots ,x_{m1},y_{1},\cdots ,x_{1M},\cdots ,x_{mM},y_{M}\right],p,q,C$  $p_{1}=q;\;p_{2}=\cdots =p_{m}=0;$  part=¬SUCCESS;Pool=[];Emax=1;  **while**
$p_{1}\geq 0$
**do**   $p_{2}=p-\sum_{j=1\&j\neq 2}^{m}p_{j};$ 5:  **if**
$p_{2}>q$
**then**    $p_{2}=q;$   **end if**   **while**
$p_{2}\geq 0$
**do**    …10:    **while**
$p_{m-1}\geq 0$
**do**     $p_{m}=p-\sum_{j=1\;\&\;j\neq m}^{m}p_{j};$     **if**
$p_{m}>q$
**then**      $p_{m}=q;$     **end if**15:        **while**
$p_{m}\geq 0\;\&\;part=\neg SUCCESS$
**do**      $A=\{p_{1},\cdots ,p_{m}\};$      part=TryPartition(A, B, C);      **if** part==SUCCESS **then**       Pool=[Pool||A];20:      **end if**      $p_{m}=p_{m}-1;$     **end while**     $p_{m}=0;$     $p_{m-1}=p_{m-1}-1;$25:    **end while**    …    $p_{3}=0;$    $p_{2}=p_{2}-1;$   **end while**30:    $p_{2}=0;$   $p_{1}=p_{1}-1;$  **end while**  **for**
i∈Pool
**do**   [RMSE, *F*]=Optimization(i, B);35:    **if**
RMSE<Emax
**then**    Emax=RMSE;    L=i;   **end if**  **end for**40:   **return**
*L*, *F*

### 2.2. Trusted Sensing Based on AEGIS and PUF

The virtual measurement, *y*, is encrypted and authenticated by using the AEGIS algorithm, which is a dedicated authentication encryption algorithm. AEGIS is based on the AES encryption round function, providing the advantage of a computational cost about half that of AES [23]. If the nonce is not reused (which should be the case for a true nonce), AEGIS provides a high security since state and key can only be recovered by exhaustive search.

AEGIS algorithm takes the cryptographic key, key, a number used only once, nonce, and a plaintex, in this case the virtual measurement, *y*, and provides a ciphertext, $C_{t}$, and an authentication tag, tag.

(2) $$\left[C_{t},tag\right]=\mathrm{AEGIS}\left(key,nonce,y\right)$$

An interesting feature of the proposed sensor is that the secret key, is not stored in the IC but it is recovered whenever needed. From a security point of view, a secret that is not stored is much more difficult to discover. Among the wide number of PUFs proposed in the literature, SRAM-based PUFs [27] are selected in this work since the proposed IC sensor requires SRAMs for its virtual sensing functionality.

**Figure 2:**
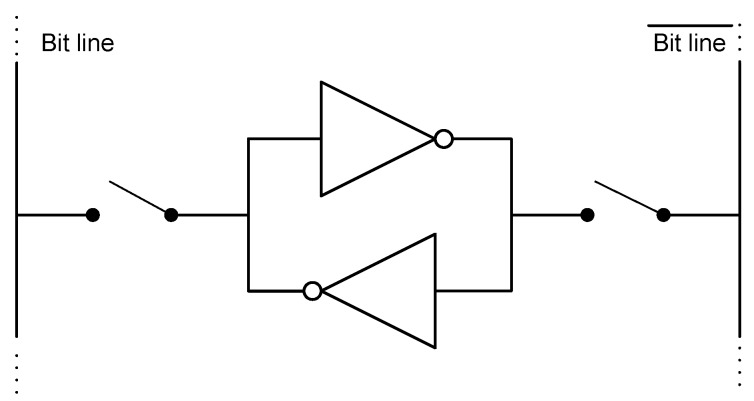
Schematic of a SRAM cell.

Since an SRAM cell is composed of two cross-coupled inverters, as shown in Figure 2, the start-up value is imposed by the inverter which begins to conduct. The conditions that make one inverter be the winner can be intrinsic or external. Intrinsic conditions are related to mismatching between the inverters, while external conditions are aging, ambient temperature, power supply voltage value ($V_{dd}$) or ramp-up time (i.e., the time to reach $V_{dd}$ after power-on) [27,28,29]. As discussed in [27], there are SRAM cells whose intrinsic conditions dominate over the external conditions. Hence, although the external conditions change, their start-up values are mostly the same. This type of cells will be named herein as ID cells. There are also SRAM cells whose external conditions dominate over the intrinsic conditions so that they are able to extract the noise of the external conditions as a source of entropy. They will be named herein as RND cells.

SRAM ID cells are used in the proposed IC sensor to recover the cryptographic key. The reliability of these stable cells is measured by the maximum fractional Hamming distance, $max_{\mathrm{ID},\mathrm{HD}}$, between pairs of responses, *R*, generated by the same *n* cells at *m* different times:

(3) $$max_{\mathrm{ID},\mathrm{HD}}=\max_{\begin{array}{c} i=1,\ldots ,m-1\\ j=i+1,\ldots ,m \end{array}}\left\{\frac{\mathrm{HD}\left(R_{i},R_{j}\right)}{n}\right\}$$

Ideally, $max_{\mathrm{ID},\mathrm{HD}}$ is zero but some bit flipping of the start-up values is unavoidable.

The SRAM cells are classified by using Algorithm 2 to obtain an ID_mask and a RND_mask. The SRAM is powered-up and down several times under different operating conditions.

HDA is used to obfuscate and to recover the key [25]. It is based on an error correcting repetition code whose correction capability should to be able to correct the maximum bit flipping probability of $max_{\mathrm{ID},\mathrm{HD}}$ in (3). Algorithm 3 describes how Helper Data, *H*, are generated from a secret key and the start-up values of the SRAM.

**Algorithm 2** Pseudo-code of Masks Extraction algorithm.**Require:** Number, J, of conditions to evaluate. Number of measurements, K, per condition  **for** i=1 to J **do**      **for** j=1 to K **do**          power down and up the SRAM          **if** j=1 **then**             save the start-up values          **else**             compare the start-up values with the stored ones          **end if**          **for** all the cells of SRAM **do**             **if** cell value does not change **then**                 add cell to ${\mathrm{ID}\_\mathrm{mask}}_{i}$             **else**                 add cell to ${\mathrm{RND}\_\mathrm{mask}}_{i}$             **end if**          **end for**      **end for**  **end for**  **for** all the cells of SRAM **do**      **if** cell belongs to all $\left\{{\mathrm{ID}\_\mathrm{mask}}_{1},\ldots ,{\mathrm{ID}\_\mathrm{mask}}_{J}\right\}$
**then**          add cell to ID_mask      **else if** cell belongs to all $\left\{{\mathrm{RND}\_\mathrm{mask}}_{1},\ldots ,{\mathrm{RND}\_\mathrm{mask}}_{J}\right\}$
**then**          add cell to RND_mask      **end if**  **end for**  **return** ID_mask, RND_mask

**Algorithm 3** Pseudo-code of Helper Data Generation algorithm.**Require:** $key=\left[k_{1},\ldots ,k_{a}\right]$, start-up values of SRAM  Each bit of the key is repeated *r* times ⟹$key_{r}=\left[k_{11},\ldots ,k_{1r},\ldots ,k_{a1},\ldots ,k_{ar}\right]$.  R= concatenation of the a·r SRAM ID cells start-up values at Helper Data generation step.  $H=key_{r}⊕R$.  **return**
*H*

The secret key is recovered as described in Algorithm 4. A response, $R^{\prime }$, slightly different to *R*, since even ID cells may provide some bit flipping, is obtained by concatenating the new start-up values of the a·r SRAM ID cells used to generate the Helper Data. The key is recovered by using an ECC with codeword length *r*, employed to correct up to $\left\lfloor \frac{r}{2}\right\rfloor$ errors.

**Algorithm 4** Pseudo-code of Key Recovering algorithm.**Require:** *H*, start-up values of SRAM  $R^{\prime }$= concatenation of the a·r SRAM ID cells start-up values at key recovering step.  $key^{\prime }=H⊕R^{\prime }=key_{r}⊕R⊕R^{\prime }$  $key=ECC(key^{\prime })$.  **return**
key

Helper Data do not reveal anything about the cryptographic key because the start-up values of SRAM ID cells obfuscate it. Similarly, Helper Data do not reveal anything about the intrinsic nature of the IC sensor because the cryptographic key obfuscates it. A way to measure the non-sensitiveness of Helper Data is to evaluate the average fractional Hamming distance, $avg_{\mathrm{InterHD}}$, between all the possible pairs of Helper Data of *p* different sensors that use the same key, as follows:

(4) $$avg_{\mathrm{InterHD}}=\frac{2}{p\left(p-1\right)}\sum_{i=1}^{p-1}\sum_{j=i+1}^{p}\frac{\mathrm{HD}\left\{R_{i},R_{j}\right\}}{n}$$

This way, instead of storing the key, only Helper Data are stored and the integrity of the sensor is ensured. If the sensor is copied, its SRAM ID cells will have other intrinsic features and they will not be able to recover the key.

The start-up values of the SRAM cells are also exploited to generate the nonce required by AEGIS. The nonce is initialized by the start-up values of SRAM RND cells and then updated by a counter. A way to measure the change of the nonce seed is to evaluate the minimum fractional Hamming distance, $min_{\mathrm{RND},\mathrm{HD}}$, between pairs of responses, *R*, generated by the same *n* RND cells at *m* different times:

(5) $$min_{\mathrm{RND},\mathrm{HD}}=\min_{\begin{array}{c} i=1,\ldots ,m-1\\ j=i+1,\ldots ,m \end{array}}\left\{\frac{\mathrm{HD}\left(R_{i},R_{j}\right)}{n}\right\}$$

A value of $min_{\mathrm{RND},\mathrm{HD}}$ strictly greater than zero ensures that the nonce seed is time variant, as required.

AEGIS is based on the AES round function [23]. There are three variations. AEGIS-128 processes a 16-byte message block with five AES round functions in parallel. It consumes the least resources, but it is the slowest for large messages. AEGIS-128L processes a 32-byte message block with eight AES round functions in parallel. In terms of resource consumption, it is the biggest version, but it is also the fastest. Finally, AEGIS-256 processes a 32-byte message block with six AES round functions. Then, it offers an intermediate performance in terms of resource consumption and time response. Since the virtual measurement is smaller or equal to 128-bits, only one 16-byte state has to be processed. Hence, AEGIS-128 is more adequate in this application, since it is as fast as AEGIS-128L, using less resources.

AEGIS-128 uses a 128-bit key and a 128-bit nonce. The algorithm is based on the function $S_{i+1}=StateUpdate\left(S_{i},m_{i}\right)$ described in Algorithm 5 where the function AESRound(A) is the AES encryption round function being *A* the state. The main operations in the AES round are SubBytes operation, ShiftRows and AES-MixColumns.

**Algorithm 5** State Update function.1: **function**
StateUpdate($S_{i},m_{i}$)2:     $S_{i+1,0}=$AESRound$\left(S_{i,4}\right)⊕S_{i,0}⊕m_{i}$3:     $S_{i+1,1}=$AESRound$\left(S_{i,0}\right)⊕S_{i,1}$4:     $S_{i+1,2}=$AESRound$\left(S_{i,1}\right)⊕S_{i,2}$5:     $S_{i+1,3}=$AESRound$\left(S_{i,2}\right)⊕S_{i,3}$6:     $S_{i+1,4}=$AESRound$\left(S_{i,3}\right)⊕S_{i,4}$7:     **return**
$S_{i+1}$8: **end function**

The pseudo-code of the AEGIS-128 applied to the virtual measurement is described in Algorithm 6. The main steps of the algorithm are to initialize the state (line 6.1), process the nonce (line 6.6), process the measurement and generate the ciphertext (line 6.14), and generate the authentication tag (line 6.20). *constant1* and *constant2* are two 128-bit constants fixed by the algorithm.

**Algorithm 6** Pseudo-code of AEGIS algorithm.**Require:** key, nonce, *y* 1: $S_{-10,0}=$ key ⊕ nonce 2: $S_{-10,1}=$ constant1 3: $S_{-10,2}=$ constant2 4: $S_{-10,3}=$ key ⊕ constant1 5: $S_{-10,4}=$ key ⊕ constant2 6: **for**
i=−10:−1
**do** 7:     **if**
*i* is odd **then** 8:         $m_{i}=$ key ⊕ nonce 9:     **else**10:         $m_{i}=$ key11:     **end if**12:     $S_{i+1}=StateUpdate\left(S_{i},m_{i}\right)$13: **end for**14: $C_{t}=y⊕S_{0,1}⊕S_{0,4}⊕\left(S_{0,2}\&S_{0,3}\right)$15: $S_{1}=StateUpdate\left(S_{0},y\right)$16: $tmp=S_{1,3}⊕128$17: **for**
i=1:6
**do**18:     $S_{i+1}=StateUpdate\left(S_{i},tmp\right)$19: **end for**20: $tag=S_{7,0}⊕S_{7,1}⊕S_{7,2}⊕S_{7,3}⊕S_{7,3}$21: **return**
$C_{t}$, tag

## 3. Architectural Description of the Sensor

The proposed sensor has two main behavioural modes. The configuration mode is carried out whenever the sensor is powered up. It recovers the key, generates a seed for the nonces, and establishes the relation between the input data and the data to estimate, that is, the PWAR model. The trusted sensing mode is set once the configuration mode is finished. It generates a PWAR virtual measurement which is encrypted and authenticated.

**Figure 3:**
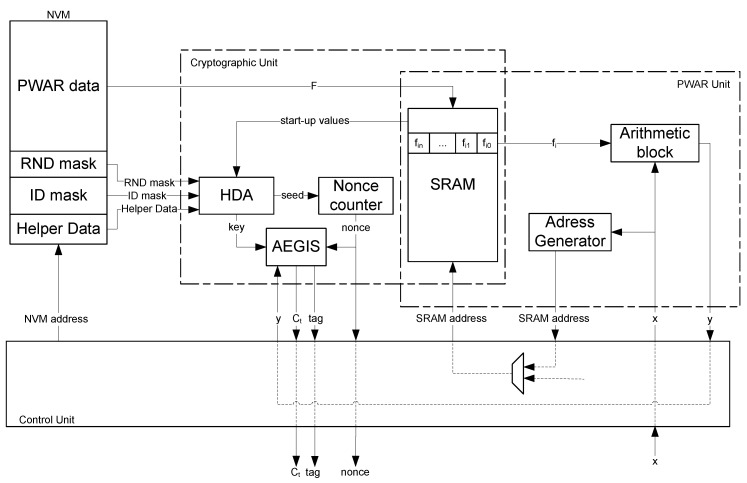
Architectural scheme of the proposed CMOS sensor.

The proposed sensor is composed of three main units: the PWAR, the Cryptographic, and the Control units. Figure 3 illustrates the block diagram of the sensor architecture, including the main signals and buses that interconnect the blocks. Note that the SRAM is shared by two units, thus saving resources since it is not used simultaneously by both units.

The non-volatile memory (NVM) should be programmed by the sensor manufacturers or their authorized distributed channels prior to use the sensor. The NVM stores the information required by the sensor: (a) the partition *L* and the parameters *F* that fix the PWAR model to implement (obtained by applying Algorithm 1); (b) the RND and ID masks (obtained by classifying the SRAM cells as exposed in Algorithm 2); and (c) the Helper Data needed to recover the cryptographic key (generated as in Algorithm 3).

The whole design prioritizes a short response time, using parallel processing, and a low power consumption, enabling the blocks only whenever needed.

### 3.1. PWAR Unit

The architecture of this unit, based on the one exposed in [30], is composed of the SRAM, the Address Generator, and the Arithmetic blocks.

Each word in the SRAM stores the coefficients of the input and the offset of the affine function associated with an hyper-rectangle, $f_{i}\in F$. Therefore, the width of the memory is $\left(n+1\right)\cdot n_{in}$ bits and the depth is the maximum number of hyper-rectangles, *P*, being $n_{in}$ the number of bits used to represent each coefficient of the input and the offset. During the trusted sensing mode, one word of the SRAM is read providing in parallel the parameters needed to compute the affine function related to an hyper-rectangle. In the configuration mode, all the PWAR parameters, *F*, are read from the NVM and stored in the SRAM.

**Figure 4:**
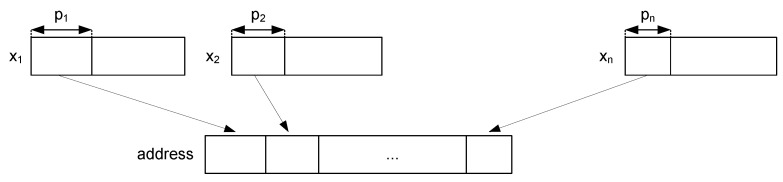
Generation of the SRAM address.

The Address Generator block is only enabled during the trusted sensing mode. It determines the address of the SRAM where the parameters associated with the input are stored. For that purpose, it concatenates the $p_{k}$ most significant bits (MSBs) of each input $x_{k}$, as shown in Figure 4. The MSBs determine which of the $2^{p_{k}}$ intervals the input belongs to, and, hence, they determine the hyper-rectangle.

As the previous block, the Arithmetic block is only enabled during the trusted sensing mode. The Arithmetic block computes an affine function for a given input *x*, and for given parameters, $f_{i}$. The operation $y=\sum_{j=1}^{n}f_{ij}\cdot x_{j}+f_{i0}$ is carried out in parallel with *n* multipliers and 1 adder. It uses fixed-point logic. The length of the fixed part is configured externally (it is one of the configuration parameters stored in the NVM).

### 3.2. Cryptographic Unit

The ID_mask, RND_mask and the Helper Data stored in the NVM are non-sensitive data. The cryptographic key, which is the sensitive information, cannot be recovered without the start-up values of the SRAM ID cells. Hence, hardware-based security is added to cryptographic-based security. During the configuration mode, both masks and the Helper Data are loaded from the NVM. During the configuration mode, the start-up values of the words in the lower part of the SRAM are read by the Cryptographic Unit. Afterwards, these cells are written with the PWAR parameters. The Cryptographic Unit is composed of the HDA block, the Nonce Counter, and the AEGIS block.

**Figure 5:**
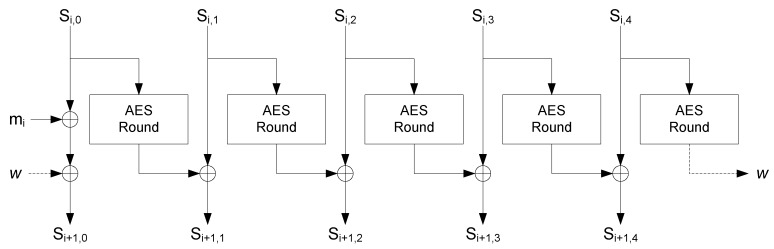
State Update.

The HDA block is only enabled during the configuration mode. It regenerates the key by using the Helper Data, the ID_mask and the start-up values of the SRAM, as described in Algorithm 2. It also provides the seed for the nonces by taking the start-up values of the SRAM cells indicated by the RND_mask. The seed is the input of a counter that is enabled whenever a nonce is required in the trusted sensing mode. During the trusted sensing mode, the AEGIS block initializes the state serially, processes the nonce, and generates both the ciphertext and the tag, as explained in Algorithm 3. The State Update operation is implemented in parallel as well as the AES round included on it. Figure 5 illustrates the implementation of the State Update.

### 3.3. Control Unit

**Figure 6:**
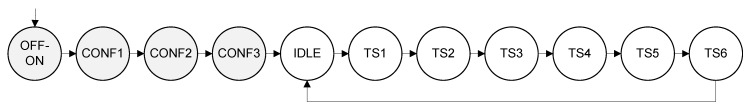
State diagram of the Control Unit.

The Control Unit copes with the two possible behavioural modes. It is implemented as a finite state machine (FSM). Figure 6 illustrates the states and the main operations. When the FSM is set in one state, only the required blocks are enabled, thus saving power consumption. The gray states correspond to the configuration mode, while the white states corresponds to the trusted sensing mode. Whenever the sensor is powered up, the configuration mode starts.

The main operations in the configuration mode are as follows. The mask_ID, mask_RND, and Helper Data are read from the NVM (CONF1). The ID and the seed are generated (CONF2). The key is recovered, the nonce is initialized with the seed, configuration data of the PWAR are read from the NVM, and the PWAR Unit is configured (CONF3).

Once the configuration mode is finished, the FSM is set to state IDLE, where it remains until the sensor receives an input data to generate a new trusted PWAR virtual measurement.

In the trusted sensing mode, the main operations are the following. Input data (*x*) are read and a new nonce is generated (TS1). The Address Generator block generates the address and the AEGIS block processes the nonce (TS2). The SRAM provides the parameters stored in the address previously generated (TS3). The PWAR Unit generates the virtual measurement (TS4). The AEGIS block processes the virtual measurement and provides the encrypted virtual measurement (TS5), and finally the AEGIS block generates the authentication tag (TS6). Once the trusted virtual sensor output is provided, the FSM goes to the state IDLE and waits for a new input data.

If the sensor is powered down and then it is powered up, the FSM starts from the state OFF-ON and all the configuration states are carried out again. Consequently, a correct performance is ensured after (expected or unexpected) suspension of power supply since the sensor is always configured before entering the trusted sensing mode.

## 4. Implementation Results

The trusted virtual sensor was synthesized in a 90-nm CMOS technology provided by Taiwan Semiconductor Manufacturing Company (TSMC). A standard low-power dual-port SRAM IP module was employed for the SRAM. The register-transfer level specifications of the other blocks were synthesized using Design Vision tool from Synopsys. The Place and Route tool used was SoC Encounter from Cadence. ModelSim simulator from Mentor Graphics was used for timing simulations.

### 4.1. Features of the Design

The non-volatile memory (NVM) has an output bus with 12 bits, so that a 12-bit word can be read in a clock cycle. It stores: (a) 20,483 12-bit words (20,480×12=4096×5×12 bits defining the affine functions and 3×12 bits defining the PWAR Unit configuration); (b) 155 12-bit words associated with the RND_mask; (c) 360 12-bit words associated with the ID_mask; and (d) 310 12-bit words (3720 bits) associated with the Helper Data.

Concerning the PWAR Unit, the maximum number of input variables, *n*, is 4. The bits of both the input variables $\left\{x_{1},\ldots ,x_{4}\right\}$ and the parameters of the affine functions are 12. The bits of the output variable, *y*, are 26. The maximum number of hyper-rectangles, $P=2^{p}$, is 4096 (p=12), and the maximum number of intervals per input, $Q=2^{q}$, is 128 (q=7). Hence, the SRAM has 4096×60 bits.

Concerning the Cryptographic Unit, the key and the nonce have 128 bits. To recover the key, a binary repetition code with codeword length r=29 is employed. Therefore, the ID size and the H size is 128×29=3712 bits. The seed of the nonce has the same size as the nonce, that is, 128 bits.

The timing responses of the main operations in both behavioural modes are detailed in the following.

The RND_mask (1860 bits) is read from the NVM in 155 clock cycles. To generate the seed of the nonce, 31 words of the SRAM (31×60=1860 bits) are read and they are selected or not for the 128-bit seed as indicated by the RND_mask. The seed generation takes 1860 clock cycles. The initialization of the Nonce Counter takes 1 clock cycle.

The ID_mask (4320 bits) is read from the NVM in 360 clock cycles. The Helper Data (3720 bits) are read in 310 clock cycles. To generate the response of 3720 ID cells, 72 words of the SRAM (72×60=4320 bits) are read and they are selected or not as indicated by the ID_mask. This takes 4320 clock cycles. The decoding to recover the secret key takes 5 clock cycles. The AEGIS needs 4 clock cycles to process the key. The PWAR configuration data and parameters are read in 3+4096×5=20,483 clock cycles.

To generate the PWAR virtual measurement, the input data are read in four clock cycles, the address of the SRAM is generated in one clock cycle, the read of a word from the SRAM takes one clock cycle, and the computation of the PWA function takes another one. Therefore, seven clock cycles are needed. Then, the AEGIS processes the nonce in 10 clock cycles. It processes the PWAR virtual measurement and provides the ciphertext in one clock cycle. Finally, the AEGIS block needs nine clock cycles to provide the authentication code. Hence, the AEGIS block takes 20 clock cycles to process the PWAR virtual measurement.

**Figure 7:**
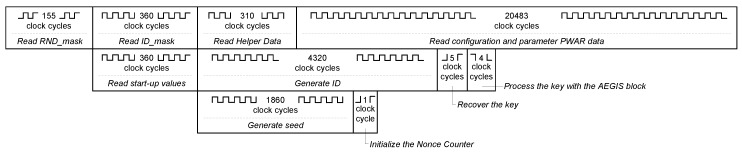
Timing of configuration mode.

The configuration mode lasts the time that the NVM is read, since the other operations can be executed in parallel. Figure 7 shows how the parallelization is carried out. Then, it lasts at most 155+360+310+20,483=21,308 clock cycles.

**Figure 8:**
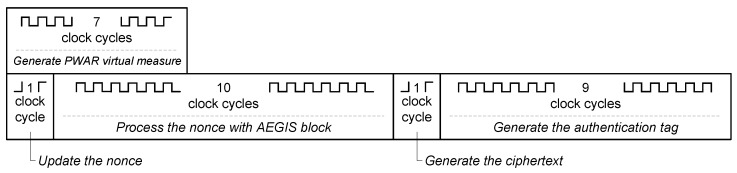
Timing of trusted virtual sensing mode.

Figure 8 shows the timing of the trusted sensing mode, it lasts 1+10+1+9=21 clock cycles.

Since the maximum operation of the trusted virtual sensor is 85 MHz, it can achieve response times lower than 0.25 microseconds.

Table 1 shows the power consumption during trusted sensing mode and the area of the main blocks provided by Design Vision tool from Synopsys.

The trusted virtual sensor occupies 0.86 mm${}^{2}$ and consumes 7.12 mW at 50 MHz. The area occupied by the Cryptographic Unit is 17.1% and its power consumption is 14.8% (considering that the SRAM and the Control are needed by virtual sensing and, hence, re-used by Cryptographic Unit).

**Figure 9:**
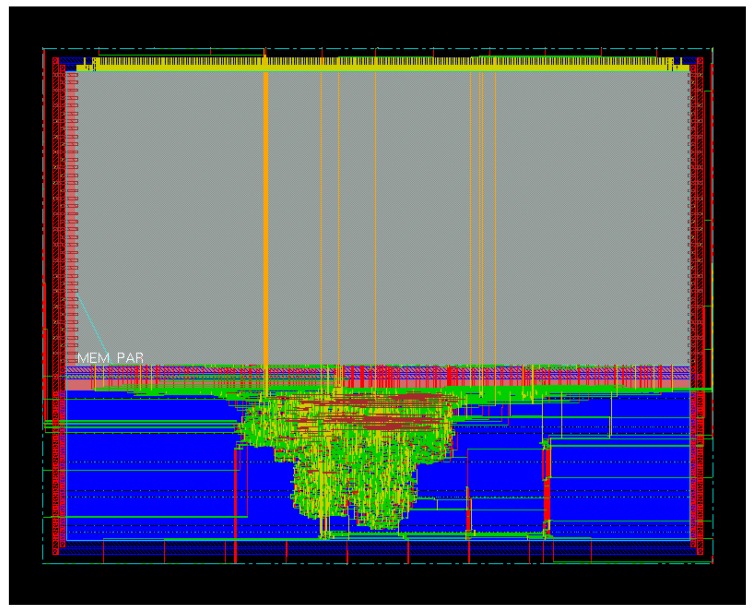
Layout of the trusted virtual sensor.

Figure 9 shows the layout of the trusted virtual sensor extracted with the SoC Encounter tool from Cadence. The largest gray rectangular area in the upper part of the layout is the SRAM.

### 4.2. Implementation Results Concerning Virtual Sensing

To evaluate the capability of the IC prototype as virtual sensor, the PWAR Unit was fabricated in TSMC 90 nm technology and verified experimentally. As illustrative example, a problem from the automotive domain, the estimation of vehicle yaw rate, was selected to be solved. In literature, this problem was firstly addressed with a silicon micromachining (MEMS)-based sensor in [31]. Since MEMS-based sensors have inaccuracy problems including severe DC-offset, an additional module based on fuzzy logic was proposed in [32]. Lately, neural-network-based direct virtual sensors implemented in software were proposed in [6]. A PWAR-based model was found for the proposed IC prototype by using Algorithm 1. The set of input-output data needed to apply the algorithm (training and test subsets) was obtained from simulations of the vehicle modelled by the differential equations described in [6], discretized with a zero-order-hold approach with a sampling time of 10 ms, and including noise in the simulated outputs (lateral acceleration and yaw rate) as in [6]. The input variables are the longitudinal vehicle speed, $v_{x}\left(t\right)$, the steering angle, $\alpha_{s}\left(t\right)$, and the lateral acceleration, $a_{y}\left(t\right)$. After applying PWAR virtual sensor algorithm, Algorithm 1, (with C=80%), the best partition found divided the longitudinal speed, the steering angle, and the lateral acceleration into 4, 16 and 2 intervals, respectively. Figure 1 shows the yaw rate provided by the PWAR-based model for a fixed value of the longitudinal speed. Therefore, the sensor estimates the yaw rate as $\dot{\Psi }=f_{i0}+f_{i1}\cdot v_{x}+f_{i2}\cdot \alpha_{s}+f_{i3}\cdot a_{y}$ if $\left(v_{x},\alpha_{s},a_{y}\right)\in P_{i}$, with i=1,…,128. The RMSE values of the PWAR-based sensor and the direct virtual sensor based on neural networks, DVS1 in [6], using simulated data, are shown in Table 2. The RMSE is lower for the PWAR-based virtual sensor.

**Figure 10:**
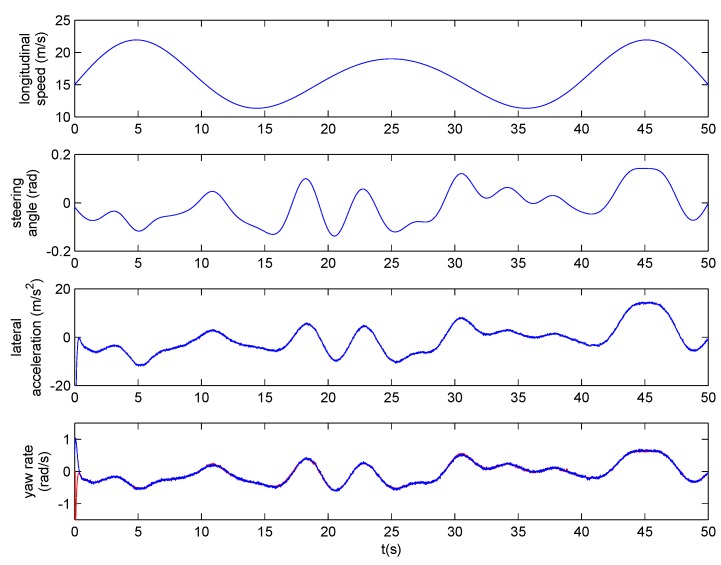
Hardware-in-the-loop simulation of the PWAR Unit.

A hardware-in-the-loop simulation was carried out in ModelSim. The evolution of the longitudinal vehicle speed, the steering angle, and the lateral acceleration scaled to their real values, are shown at the upper part of Figure 10. The yaw rate estimated by the sensor (also scaled to the real value) is shown in blue at the bottom of Figure 10. The yaw rate obtained by the model is shown in red. Noise can be observed in the lateral acceleration and the yaw rate of the model that makes it more realistic. To achieve a response time lower than the 10 ms sampling time, the sensor should work at more than 2.1 KHz.

### 4.3. Implementation Results Concerning Trusted Sensing

Since the behaviour of the SRAM is fundamental to ensure the trusted sensing mode of the prototype, the SRAM was fabricated in TSMC 90-nm technology and analyzed experimentally. Ten samples of 4096×60 SRAMs were measured as follows. The cells of the SRAM were classified three times after 20 measurements each time by using Algorithm 2 with J=3 and K=20. Nominal operation conditions were considered three times: power supply $V_{dd}=1.2V$ and temperature *T* = 25 °C. The $V_{dd}$ was supplied to the SRAM with a measured ramp-up time of 18.2 ms. The average of ID cells found in percentage was 87.27±0.23% and that of RND cells was 7.15±0.16%. To obtain an ID of 3760 bits, as required, 72 words of 60 bits are enough since 3770 bits (ID cells) are obtained in average from 4320 SRAM cells. To obtain a seed for the nonces of 128 bits, 31 words of 60 bits are enough. On average, the 7.15% of 31×60=1860 cells are 132 RND cells.

To evaluate the robustness of the key recovering in the designed prototype, Algorithm 2 was applied considering different operating conditions (with J = 2 and K = 20): (a) changing the value of $V_{dd}$ to 1.08V and to 1.32V (±10% of the nominal value); (b) changing the value of T to 5 °C and 75 °C; (c) after accelerated aging the ICs with the SRAM continuously during 171 h at high temperature of 75 °C (and nominal $V_{dd}$); and (d) decreasing the $V_{dd}$ ramp-up time to 2.32 ms.

The first row of Table 3 shows the average of maximum intra HD (3) between responses of SRAM ID cells taken at operation conditions with changes in $V_{dd}$, *T*, ramp-up time, and aging (before and after aging). Reliability is mostly affected by changes in ramp-up time and temperature. Since the start-up values of different SRAM ID cells of the same SRAM were proved to be independent, the probability that a string of 29 bits contains more than 14 errors (and, hence, it is not correctly decoded to extract the original key bit) is calculated with the following formula:

(6) Pbit−error=1−∑i=01429ipi·1−p29−i

Assuming the worst-case bit flipping probability in the first row of Table 3, p=9.64%, the $P_{\mathrm{bit}-\mathrm{error}}$ is $1.19\times 10^{-8}$. Similarly, assuming $1.19\times 10^{-8}$ as the worst-case bit-error probability in the key, the probability that the 128-bit key is not correctly decoded by the authentic sensor is $1.53\times 10^{-6}$, which is quite low. The average inter HD (4) between responses of ID cells of 10 different SRAMs (corresponding to different sensors) was measured as 0.50±0.01 in nominal conditions. As commented in Section 2.2, this result shows that Helper Data do not reveal information about the cryptographic key. Taking such average inter HD as bit flipping probability between a false sensor and a genuine one, the probability of correctly decoding one bit of the cryptographic key is 0.5, and, hence, the probability of regenerating the correct key by a false sensor is $2\times 10^{-128}$, which is completely infeasible. The second row of Table 3 shows the average of minimum inter HD (5) between responses of RND cells taken at the different operation conditions. In any case, it is ensured that the nonce seed is always different, as required.

## 5. Conclusions

The VLSI design of trusted virtual sensors is very suitable for industrial applications where security is becoming increasingly important since it offers privacy, authenticity and integrity of the virtually sensed measurement and the circuit itself. The implementation of the design into a 90-nm CMOS technology occupies 0.86 mm${}^{2}$ and consumes 7.12 mW when trusted sensing at 50 MHz. Working at maximum frequency, the trusted virtual sensor allows sampling times lower than 0.25 μs. The inclusion of security to the virtual sensor needs 17.1% of active area and 14.8% of power consumption.

## Figures and Tables

**Table 1:** Area and power consumption during trusted sensing mode.

	Area	Power@50MHz
**HDA block**	0.0028 mm${}^{2}$	0.13 mW
**Nonce Counter**	0.0041 mm${}^{2}$	0.15 mW
**AEGIS**	0.14 mm${}^{2}$	0.77 mW
**Arithmetic block**	0.015 mm${}^{2}$	0.24 mW
**Address Generator block**	0.0007 mm${}^{2}$	0.15 mW
**SRAM**	0.67 mm${}^{2}$	4.49 mW
**Control Unit**	0.025 mm${}^{2}$	1.19 mW

**Table 2:** Error comparison between virtual sensors.

	Direct Virtual Sensor in [6]		PWAR Virtual Sensor	
	**Training Data**	**Testing Data**	**Training Data**	**Testing Data**
**RMSE**	3.9%	6.5%	0.25%	1.08%

**Table 3:** Features of the SRAM PUF in the IC sensor.

	Changes in $V_{\mathrm{dd}}$	Changes in *T*	Changes in Ramp-Up Time	Changes in Aging
**$max_{\mathrm{ID},\mathrm{HD}}$ (ID)**	0.66±0.04%	2.75±0.07%	9.64±0.24%	0.29±0.01%
**$min_{\mathrm{RND},\mathrm{HD}}$ (RND)**	34.31±0.29%	41.49±0.51%	39.02±0.49%	33.74±0.31%
